# Synthesis of Nickel Nanowires with Tunable Characteristics

**DOI:** 10.3390/nano6010019

**Published:** 2016-01-15

**Authors:** Zengzilu Xia, Weijia Wen

**Affiliations:** Nano Science and Technology Program, Hong Kong University of Science and Technology, Clear Water Bay, Kowloon, Hong Kong; zxiaab@connect.ust.hk

**Keywords:** nanostructures, nanowires, nickel, long-term stability, high purity, tunable characteristics

## Abstract

A one-step synthesis of magnetic nickel nanowires (NiNWs) with tunable characteristics is reported. The method is simple and easy to be conducted, leading to high compatibility with scaling-up. It is discovered that the size and morphology of NiNWs can be adjusted by tuning the reaction temperature, time length, as well as surfactant concentration. It is found that the products have shown high purity which remained after being stored for several months. A remarkable enhanced saturation magnetization of the product was also observed, compared to that of bulk nickel. By providing both practical experimental details and in-depth mechanism, the work introduced in this paper may advance the mass production and further applications of NiNWs.

## 1. Introduction

In the view of nanomaterials, morphology and size are proved to serve a significantly important role in determining their properties, such as reactivity, opto-electronic, and magnetic properties [1,2]. Nickel (Ni) is a celebrated magnetic material, having potential applications in storage media, microwave absorption, catalysis, and clinical treatment [3,4,5,6,7]. It is remarkable that magnetic properties of Ni nanomaterial are strongly dependent to the finite-size effect, and can be varied by controlling the shape and size [8,9]. Diverse types of morphologies and sizes of Ni nanomaterials have been synthesized, including chains [10], belts [11], tubules [12], rods [13], triangular plates [14], hexagonal hierarchy [9], hollow spheres [15], particles, and flowers [16]. Among these members, nickel nanowire (NiNW) is a prominent example for having different properties from bulk metal due to the one-dimensional (1D) geometry and large shape anisotropy, which can enhance the coercivity and prevent it from becoming superparamagnetic [17,18]. Based on the special properties of NiNWs, numerous promising applications have been achieved, such as optical sensing, plasmonic resonance sensors, perpendicular magnetic storage, functional devices, negative permeability, biological separation, wire-grid type micro polarizer, trenches, and capacitors [19,20].

Different techniques generating NiNWs include, but are not limited to, chemical vapor deposition (CVD) [21], electrochemical deposition [19,22], electrospinning [23,24], bacterial system approach [25], microwave-assisted process [17,26], and solvothermal methods [27]. Despite these techniques successfully fabricating products, a further scale-up of production would be mitigated for the specialized requirements and cost, such as the templates (CVD and electrodeposition), high voltage (electrospinning), biological culture (bacterial system), high energy processing, Teflon-lined stainless steel autoclaves (solvothermal approach), or microwave generators (microwave heating). Consequently, an approach which could be conducted with little time, without catalysts or extraneous chemicals, at a relatively low temperature, under ambient pressure conditions is desirable for synthesis of NiNW with a well-defined 1D structure. To date, various groups have been focusing on this expectation and investigated several simple and mild conditions [28,29,30,31,32,33,34], yet there are still some gaps. For instance, when using sodium borohydride (NaBH_4_) as the reduction agent, boron could be absorbed into the product [30,35] and, thus, the NiNWs are no longer pure elemental metal. Meanwhile, it should be noted that sodium hydroxide (NaOH) has been widely used in NiNWs synthesis [11,36,37,38] for serving as a pH adjustor and catalyst [39]. Since it is not a necessary chemical for 1D structure formation, we would like to design the experiment without NaOH.

Therefore, the development of a facile method to synthesize high purity NiNWs is necessary and important. In this work, we synthesized highly pure NiNW with a mild and conducive reaction which does not need extraneous chemicals. These pure NiNWs showed high stability under ambient conditions. At the same time, investigations of reaction conditions for morphology controlling were conducted based on the information from X-ray diffraction (XRD), scanning electron microscopy (SEM), transmission electron microscopy (TEM), energy-dispersive X-ray spectroscopy (EDS), and vibrating sample magnetometer (VSM). Our method and the results in this article are expected to provide useful information for further NiNW researches such as biological applications, and its industrial mass production.

## 2. Results and Discussion

**Figure 1:**
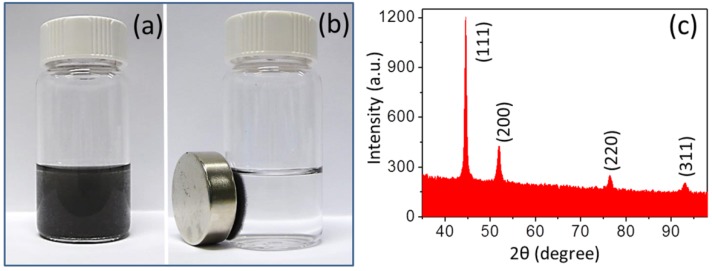

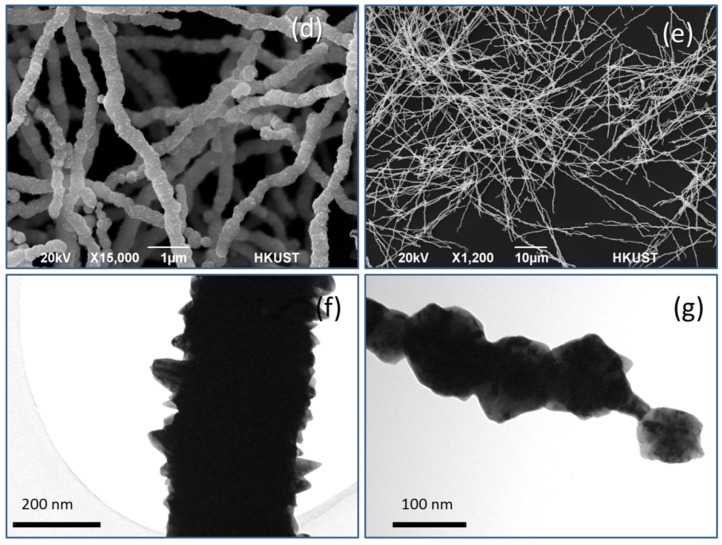
The photographs of nickel nanowires (NiNWs) (**a**) dispersed in ethanol (**b**) attracted by an external magnet, (**c**) X-ray diffraction (XRD) patterns, (**d**) low and (**e**) high magnification scanning electron microscopy (SEM) images of NiNWs; and the transmission electron microscopy (TEM) images of (**f**) the body and (**g**) the end of a NiNW.

The NiNWs synthesized with typical conditions was characterized to study its structure and morphology. The product was a dark gray solid with metallic luster, having a nice dispersibility in ethanol, and it could be sufficiently attracted by an external magnet (Figure 1a,b). The process of dispersing and aggregating was reversible. The XRD pattern peaks (Figure 1c) located at 44.5°, 51.9°, 76.5°, and 92.9° could be matched with (111), (200), (220), and (311) crystalline planes corresponding to the face-centered cubic (*fcc*) structure of Ni (Joint Committee on Powder Diffraction Standards File No. 04-0850). The XRD analysis revealed that the NiNWs were crystallized with a lattice constant of 2.037 Å. Dimensions of product were evaluated from the analysis of 50 individual NiNWs to represent the general size of the product, while the morphology would consist of the surface appearance of NW and the shape anisotropy expressed by the aspect ratio or length-to-width ratio (LWR) calculated with the mean values of length and width. SEM images (Figure 1d,e) demonstrate NiNWs having an average length of 52 ± 17 μm, an average diameter of 330 ± 60 nm, and corresponding LWR of 158 ± 58. Based on the TEM images, the body of NiNWs indicated highly uniform shape (Figure 1f). Smaller diameters appeared than the NW main body, and there were nanoparticles linked to the NiNW end (Figure 1g). Several isolated Ni nanospheres were observed existing among NWs (Figure 1d). However, the amount of NiNWs was dominant in this system, at about 99%, and the amount of Ni nanoparticles would reduce with more washing.

In order to have better control on the NiNW size and morphology in this system, investigations of the impact from three reaction parameters, namely temperature, time, and surfactant concentration, on the product were conducted.

### 2.1. Reaction Temperature Control

**Figure 2:**
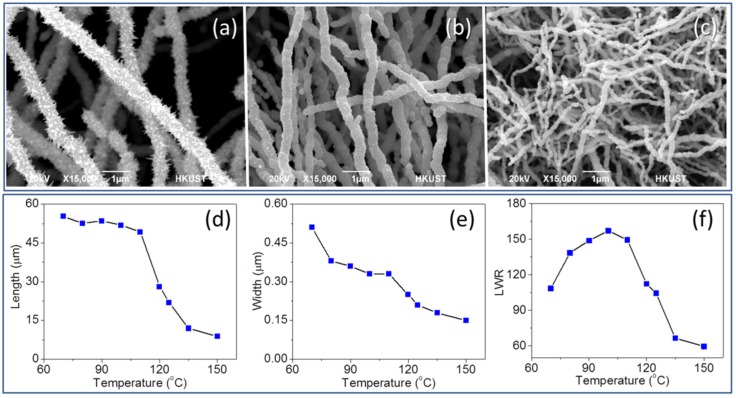
SEM images with same magnification of NiNWs synthesized at (**a**) 70 °C, (**b**) 110 °C, and (**c**) 150 °C. The change of (**d**) length, (**e**) width, and (**f**) length-to-width ratio (LWR) of NiNWs with temperature.

We found that the reaction temperature plays an important role in determining the size and morphology of NiNWs. Experiments were conducted with reaction temperature at 60, 70, 80, 90, 100, 110, 120, 125, 135, and 150 °C. SEM images indicate a continuous change of NiNW appearance with the reaction temperature (Figure 2a–c). As the temperature increased, the reaction increased in efficiency. For instance, it took about 100 min to form floating solid product at 70 °C, while only 5 min required to complete NiNW formation at 150 °C. The changes of average length, average width, and LWR *versus* the temperature were given in line charts (Figure 2d–f). The largest shape anisotropy belonged to the NWs synthesized at reaction temperature of 100 °C, and it dropped sharply when the temperature reached above 125 °C. Lower reaction temperatures lead to larger dimensions of NiNWs and palpable growth of the surface nanopricks. Although products synthesized at lower temperature should have larger dimensions than the ones synthesized at higher temperature, they usually had many NWs with short length or fragments which diminishing the average values. It should be noted that there was an obvious decrease of yield when reaction temperature was below 90 °C, and eventually no product was obtained at 60 °C. The size and number of nanopricks on the NiNW surface can sufficiently influence the surface-to-volume value. This phenomenon is worth being noted, since there are few reports focusing on promoting the growth of these nanopricks. Among the three parameters discussed in this article, low temperature is the only one which could provide an obvious furtherance to the growth of the surface nanopricks. When reaction temperature was above 90 °C, the number and size of nanopricks would decline sharply. There was no more obvious presence of surface nanopricks when the temperature was at 150 °C.

### 2.2. Reaction Time Length Control

**Figure 3:**
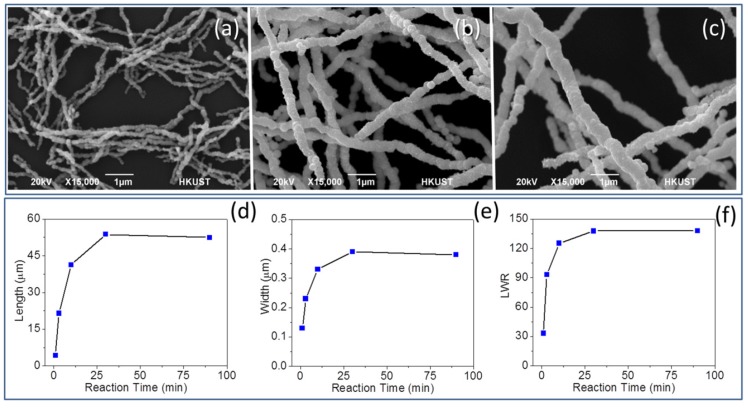
Typical SEM images with same magnification of NiNWs synthesized with (**a**) 1 min, (**b**) 10 min, and (**c**) 90 min. The change of (**d**) length, (**e**) width, and (**f**) LWR of NiNWs with reaction time.

For the investigation of time, NiNWs were prepared by conducting the experiment with a reaction time length of 1 min, 3 min, 10 min, 30 min, and 90 min, separately. According to the SEM images (Figure 3a–c), it is notable that the reaction time had a strong influence on the size of the product, yet it might not have significant effect on the formation of surface nanopricks on NiNW after 1 min. The length, as well as width, of NiNWs would increase quickly in the first 10 min, with a sudden change of the shape anisotropy (Figure 3d–f). Then the growth would slow down, and there was finally no distinct growth 30 min after the reaction started. This result is consistent with the time taken for the solid product forming and floating to the solution surface of about 30 min in a typical synthesis procedure. The sensitivity to reaction time in the first 10 min gave an advantage for controlling the NiNW dimensions, as well as the anisotropy, by simply controlling the time length. While small-sized NiNWs could be obtained with a short time, the low yield of the product would be a significant drawback in this case. The independency of NiNW size and morphology from the time after 30 min would provide a huge convenience in industrial fabrication due to a less harsh requirement for reaction time control. Meanwhile, this group of samples can be used to study the NiNW growth mechanism, which will be discussed at Section 2.5 in detailed.

### 2.3. Surfactant Concentration Control

**Figure 4:**
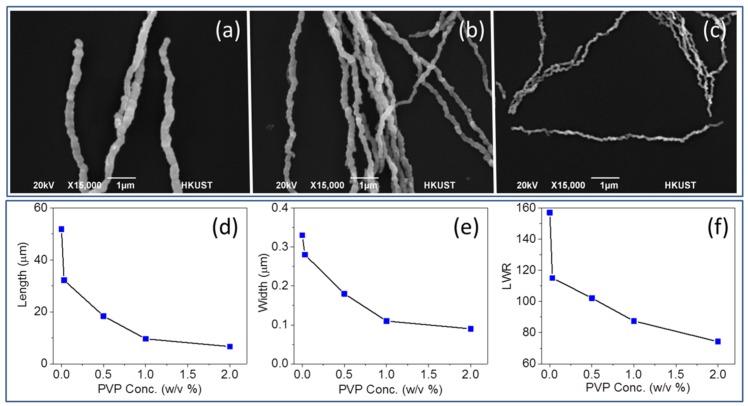
SEM images with same magnification of NiNWs synthesized with poly(vinylpyrrolidone) (PVP) concentration of (**a**) 0.03 *w*/*v* %, (**b**) 0.5 *w*/*v* %, and (**c**) 2 *w*/*v* %. The change of (**d**) length, (**e**) width, and (**f**) LWR of NiNWs with PVP concentration.

PVP served as regular surfactant in NiNW synthesis [2,40]. To investigate the influence from surfactant concentration, NiNWs were synthesized with PVP concentrations of 0.03, 0.5, 1, 2, 5, and 10 *w*/*v* % in EG, respectively (Figure 4a–c). The typical synthesized product is considered as having no PVP condition in this part of discussion. Changes of dimensions, as well as LWR *versus* PVP concentration change, were present as line charts (Figure 4d–f). Although PVP concentration showed no significant impact on the yield and reaction time length, there was no product when PVP concentrations were higher than 2 *w*/*v* %. We noted that PVP had a strong control on the diameter of NWs, which was consistent with the stabilization function of PVP on the surface of NWs. When the PVP concentration was 2 *w*/*v* %, the average width was only 90 ± 20 nm, which was the smallest dimension among all the obtained products and is the only average value less than 100 nm. PVP also served a role in reducing the shape anisotropy of NiNWs and restrained the growth of surface nanopricks. For instance, NiNWs with LWR of 74 ± 34 had no nanopricks on the surface (Figure S1). This smooth surface could not be obtained by varying temperature or reaction time, yet it was possibly caused by the strength of interaction between PVP and the NiNW surface.

### 2.4. Purity and Magnetic Properties

**Figure 5:**
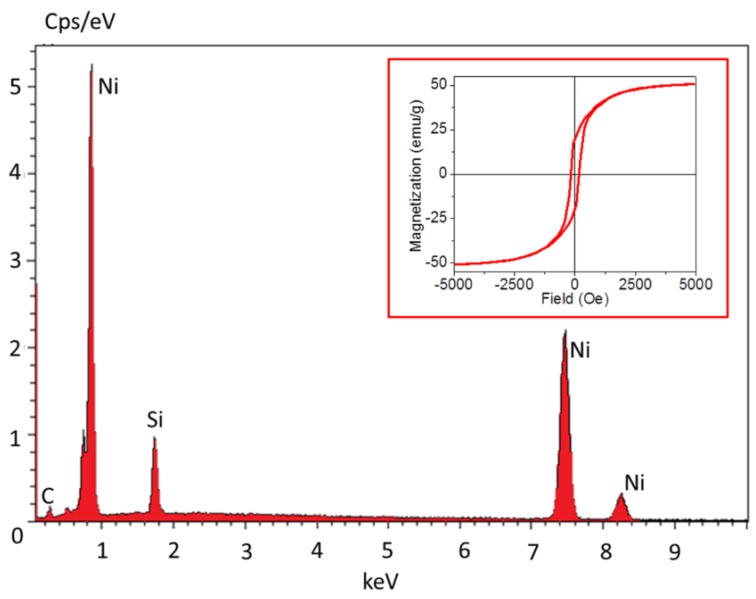
The typical energy-dispersive X-ray spectroscopy (EDS) spectrum. The inset is hysteretic loop at room temperature of typical NiNWs. These characterizations were conducted on the product synthesized with typical process.

One remarkable property of our product is the high purity which is expected to be stable over the long-term under ambient storage conditions. NiNWs synthesized with PVP addition had oxygen and nitrogen peaks in the EDS spectra, but these peaks might come from the PVP remaining on NWs (Figure S1). In addition, the noticeable carbon peak and silicon peak could be attributed to the carbon tape and underlying silicon wafer, respectively. For samples synthesized without PVP, although a small peak could be found at the characteristic position of oxygen K-series in the EDS spectra, the amount of oxygen was undetectable (Figure 5). This indicated the high purity of our products and we believed that the small amount of NiO was located on the NiNWs surface to prevent further oxidation. In fact, the typicaly synthesized product showed superior ambient storage stability. As shown in Figure S2, the high purity remained even after one month in air at room temperature, and only a small amount of oxygen was detected after five months. NiNW also kept this high purity even after a treatment at 70 °C for 30 h. Consequently, these pure NiNWs could be claimed to be inert under ambient condition with normal temperature and pressure.

Another remarkable point is the enhanced magnetic property. The saturation magnetization (*M*_s_) of bulk nickel is 55 emu/g, remnant magnetism (*M*_r_) is 2.7 emu/g, and coercivity (*H*_c_) is 100 Oe at room temperature [41]. In this system, the typically fabricated product has *M*_s_ 50.8 emu/g, *M*_r_ 19.9 emu/g, and *H*_c_ 167.7 Oe at room temperature (Figure 5). Compared to the bulk Ni, our product showed enhanced *M*_r_ and *H*_c_ properties, which would be consistent of its shape anisotropy. The bulk Ni should generally have a higher *M*_s_ than NiNW counterparts, because the total magnetic moment of magnetic nanostructures has been reduced by the surface spin disorder [37]. The larger size the product has, the closer magnetic properties approaching to the bulk material (Table S1). The The *M*_s_ value was proportional to the NiNW diameter, while the *H*_c_ showed reverse proportional to that. This result was consistent to the published report [17].

### 2.5. Reaction Mechanism

**Figure 6:**
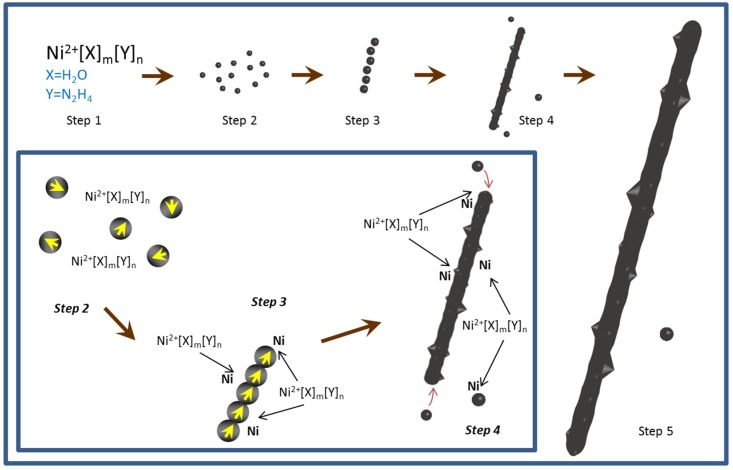
The illustration of NiNW growth mechanism. The inset is the detailed illustration of Step 2 to 4.

A plausible scheme of the NiNW growth mechanism is that a soluble complex of Ni ions and N_2_H_4_ would be formed when N_2_H_4_·H_2_O was added into the solution [27,42]. N_2_H_4_ served as a bridging bidentate ligand towards the metal center besides acting as the reduction agent [43], which caused the solution become blue. The solution changed to turbid in a very short time because this complex would be quickly reduced into a short chain-like nanostructure [37]. With Ostwald ripening and further growth, large shape anisotropy NiNWs could be obtained. The morphology and size change of time-controlled products might be considered as supporting evidence to the above statement. A supplementary hypothesis is suggested to the present scheme. Since Ni nanoparticles existed in the final product, the growth of such nanoparticles should be considered as a competitive process to the NiNW formation (Figure 6). Although other reports [30,37,38] believe that single Ni nuclei would be formed before the formation of short nanochains, the exact evidence for this stage could not be observed by our sampling (Figure S3).

## 3. Experimental Section

*Chemicals.* Nickel (II) chloride hexahydrate (NiCl_2_·6H_2_O; 99.9%), ethylene glycol (EG; 99.8%), hydrazine monohydrate (N_2_H_4_·H_2_O; 98%), and poly(vinylpyrrolidone) (PVP; *M*_w_, 40,000) were purchased from Sigma-Aldrich (St Louis, MO, USA). Ultrapure deionized water (DI; millipore water systems) with resistivity 18.2 MΩ·cm^−1^ was used throughout the work. All chemicals were used without further purification.

*Process.* In a typical procedure, 75 μL 1 M aqueous NiCl_2_ solution and 15 mL EG were mixed and heated to 100 °C, and then 0.5 mL N_2_H_4_·H_2_O was added in, dropwise. The whole mixture was maintained at this temperature for about 30 min until the dark gray product was formed and eventually floated at the solution surface. The dark gray product was then washed with DI water and anhydrous ethanol several times by magnetic decantation, and then dispersed in ethanol for further characterization. For PVP-controlled conditions, specific concentration of PVP in EG solution was used instead of pure EG as the solvent in this system.

*Characterization.* The XRD pattern was recorded on PANalytical X’pert Pro diffractometer (Cukα_1_ radiation, λ = 1.540562 Å, 40 kV, 40 mA, Almelo, Netherlands). SEM and TEM images were obtained with JEOL JEM6390 SEM and JEOL JEM100CXII TEM (Tokyo, Japan), correspondingly. EDS patterns were obtained with the capabilities (Bruker, XFlash Detector 4010, Billerica, MA, USA) equipped on SEM. VSM measurements were conducted by LakeShore 7300 VSM (Westerville, OH, USA) at room temperature with a maximum magnetic field of 5 kOe.

## 4. Conclusions

In summary, a facile size and morphology-tunable method has been exploited to synthesize pure NiNWs with high stability under ambient storage conditions. The product was observed to be crystallized in the *fcc* phase and have large shape anisotropy over 100. NiNWs also exhibited an enhanced magnetic properties over bulk Ni due to the large shape anisotropy. The reaction parameters controlling the size and morphology, such as the reaction temperature, time, and surfactant concentration, were investigated in detail. In addition, a supplemental hypothesis was suggested to the present NiNW growth mechanism. It is expected that this method and the complementary results will be serviceable for further development on industrial scale-up fabrication and other NiNW applications.

## Supplementary Materials

They are available online at http://www.mdpi.com/2079-4991/6/1/19/s1.
