# Supplementary material for: Synthesis of Nickel Nanowires with Tunable Characteristics
Source: Nanomaterials (Basel). 2016 Jan 15;6(1):19. doi: 10.3390/nano6010019 (PMC5302550; doi:10.3390/nano6010019)
Supplement: Supplementary file 1 [file nanomaterials-06-00019-s001.pdf]

Supplementary Materials

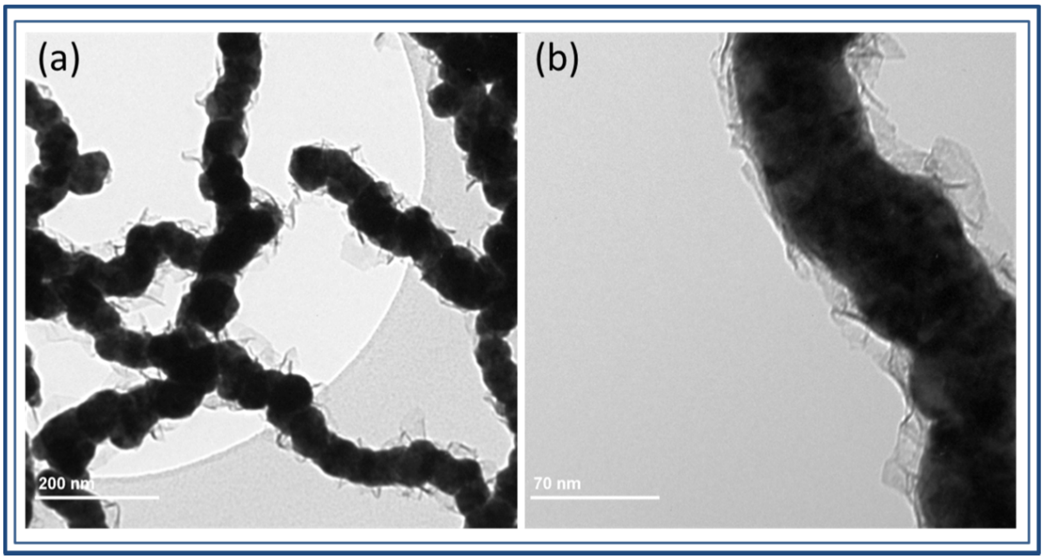

Figure S1. (**a**) The transmission electron microscopy (TEM) images of nickel nanowires (NiNWs) synthesized with 2 *w*/*v* % poly(vinylpyrrolidone) (PVP). (**b**) The higher magnification shows more clearly that there was some PVP remaining on the NiNW surface.

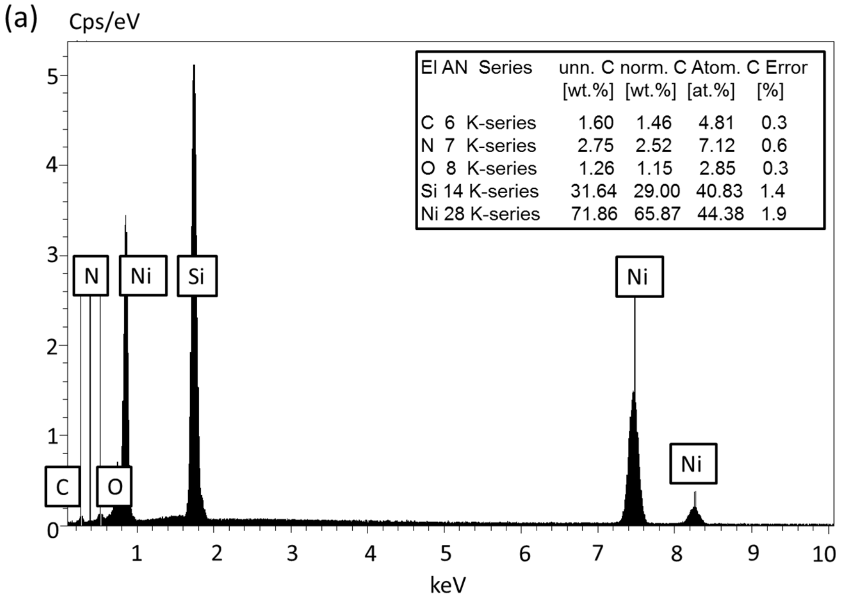

Figure S2. *Cont.*

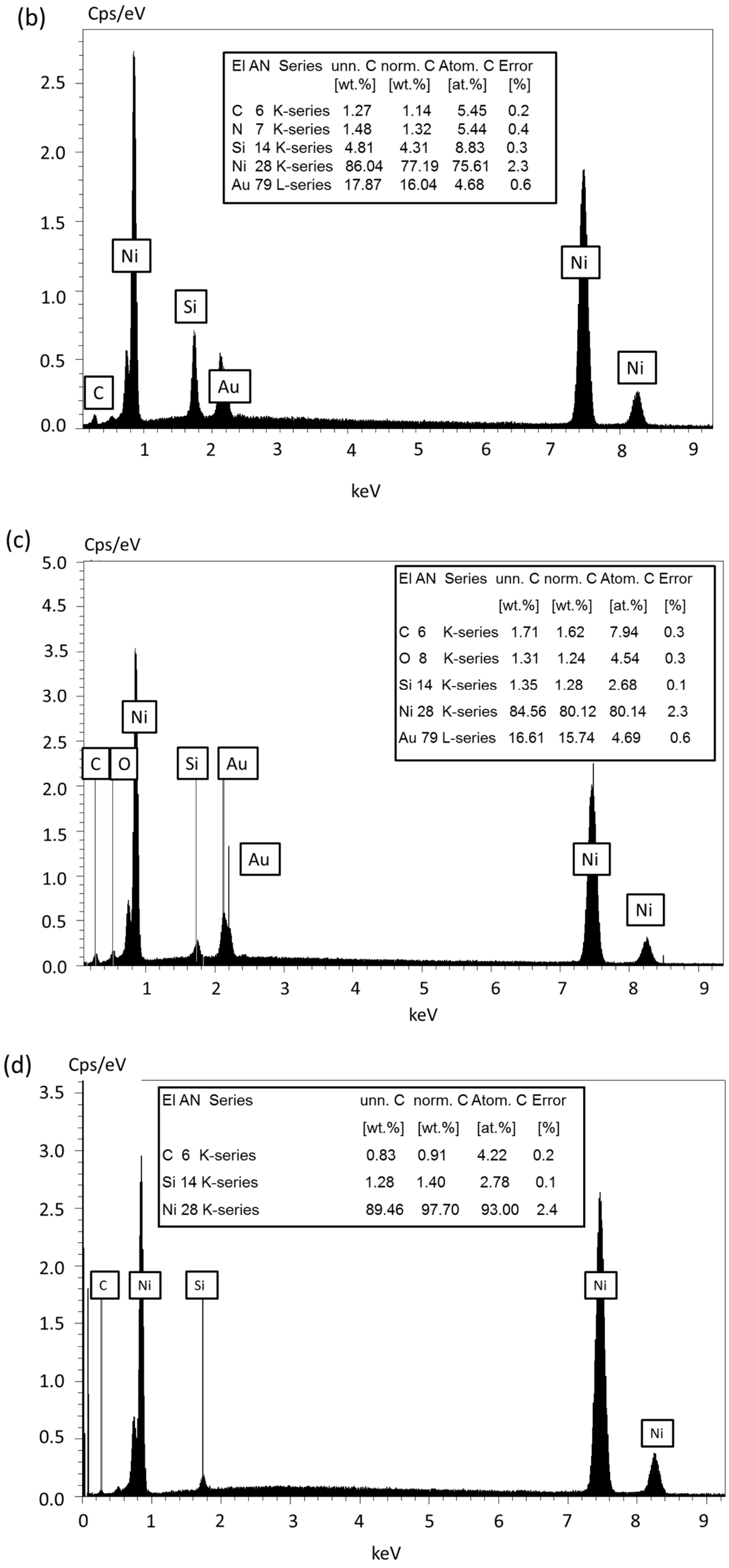

Figure S2. The energy-dispersive X-ray spectroscopy (EDS) spectrum of NiNWs (**a**) synthesized with PVP addition (**b**) under ambient condition for one month; (**c**) five months; and (**d**) with heating treatment at 70 °C for 30 h. The gold peak can be attributed to the gold layer coated to increase the resolution of the scanning electron microscopy (SEM) image.

Table S1. The vibrating sample magnetometer (VSM) comparison of magnetic properties from 3 different products with the bulk material.

Width (nm)	*Ms* (emu/g)	*Hc* (Oe)
**Bulk [1]**	55	100
**330**	50.8	167.7
**280**	49.7	179.9
**120**	39.9	185.4

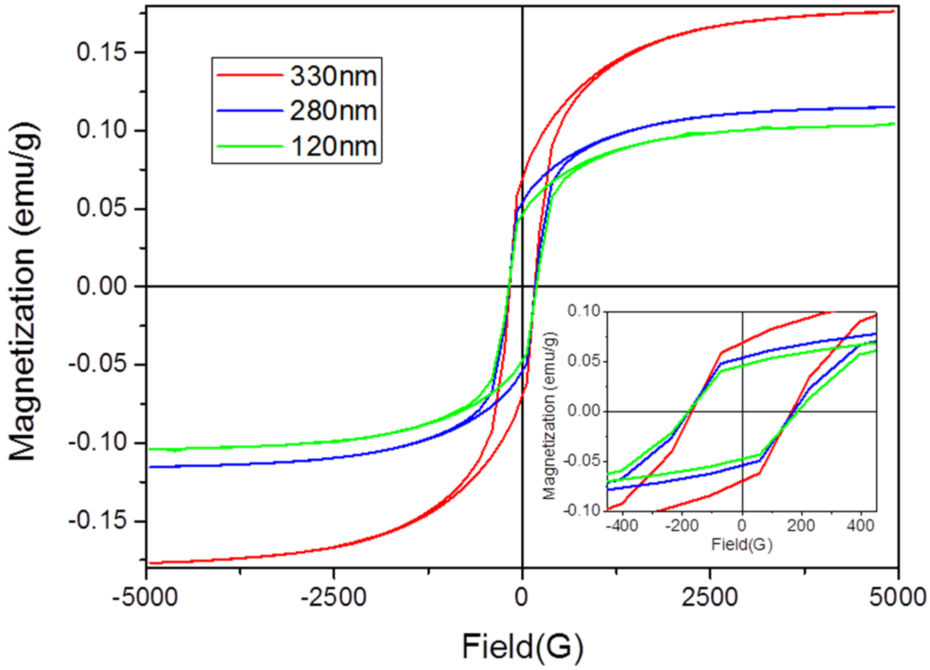

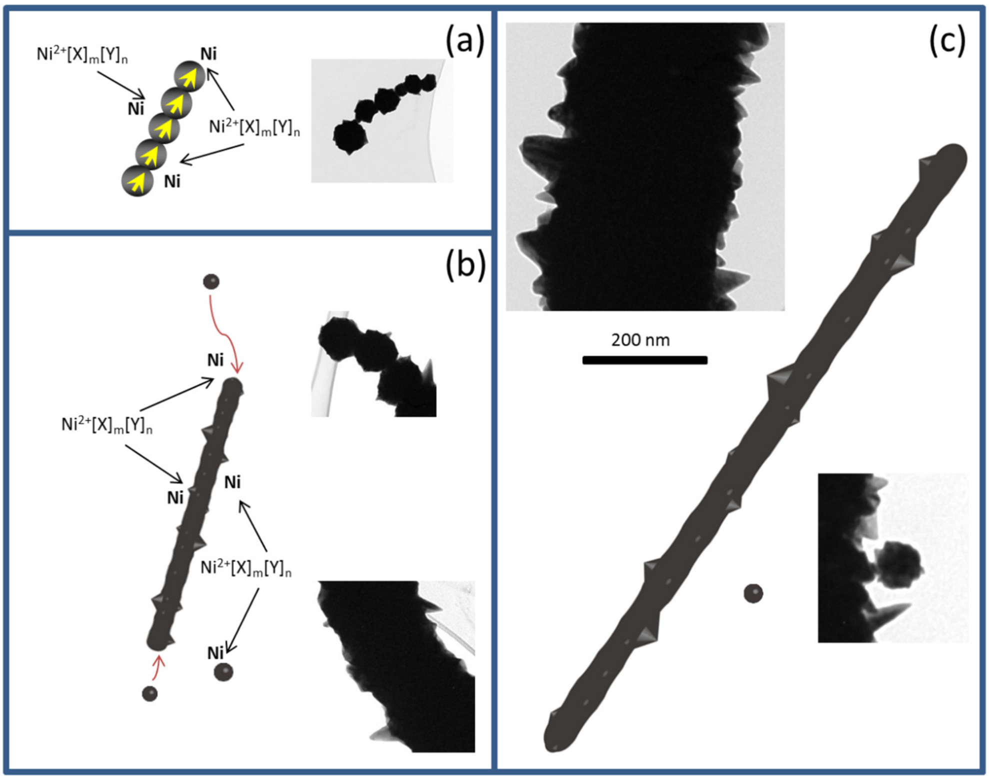

Figure S3. Corresponding TEM images to (**a**) Step 3; (**b**) Step 4; and (**c**) Step 5.

References

1. Hwang, J.H.; Dravid, V.P.; Teng, M.H.; Host, J.J.; Elliott, B.R.; Johnson, D.L.; Mason, T.O. Magnetic properties of graphitically encapsulated nickel nanocrystals. *J. Mater. Res.* **1997**, *12*, 1076–1082.

© 2016 by the authors; licensee MDPI, Basel, Switzerland. This article is an open access article distributed under the terms and conditions of the Creative Commons by Attribution (CC-BY) license (http://creativecommons.org/licenses/by/4.0/).
